# Prediction of CD4 T-Lymphocyte Count Using WHO Clinical Staging among ART-Naïve HIV-Infected Adolescents and Adults in Northern Ethiopia: A Retrospective Study

**DOI:** 10.1155/2020/2163486

**Published:** 2020-04-30

**Authors:** Abraham Desta Aregay, Kibriti Mehari Kidane, Asfawosen Berhe Aregay, Kiros Ajemu Fenta, Ataklti Gebretsadik Woldegebriel, Hagos Godefay, Tewolde Wubayehu Woldearegay

**Affiliations:** ^1^Tigray Health Research Institute, Mekelle, Tigray, Ethiopia; ^2^Tigray Regional Health Bureau, Mekelle, Tigray, Ethiopia

## Abstract

**Background:**

WHO clinical staging has long been used to assess the immunological status of HIV-infected patients at initiation of antiretroviral therapy and during treatment follow-up. In setups where CD4 count determination is not readily available, WHO clinical staging is a viable option. However, correlation between CD4 count and WHO clinical staging is not known in an Ethiopian setting, and hence, the main aim of this study was to assess predictability of CD4 T-lymphocyte count using WHO clinical staging among ART-naïve HIV-infected adolescents and adults in northern Ethiopia.

**Methods:**

A retrospective cross-sectional study was done in the Tigray Region, Ethiopia, from April 2015 to January 2019 from a secondary database of 19525 HIV-infected patients on antiretroviral treatment. Analysis was done using STATA-14.0 to estimate the frequencies, mean, and median of CD4 T-cell count in each WHO stages. Sensitivity, specificity, positive predictive value, negative predictive value, kappa test, and correlations were calculated to show the relationships between WHO stages and CD T-cell count.

**Results:**

The sensitivity of WHO clinical staging to predict CD4 T-cell counts of <200 cells/*μ*l was 94.17% with a specificity of 3.62%. The PPV was 49.03%, and the NPV was 3.62%. The sensitivity of WHO clinical staging to predict CD4 T-cell counts of <350 cells/*μ*l was 94.75% with a specificity of 3.00%. The PPV was 75.81%, and the NPV was 15.09%. Similarly, the sensitivity of WHO clinical staging to predict CD4 T-cell counts of <500 cells/*μ*l was 95.03% with a specificity of 2.73% and the PPV and NPV were 88.32% and 6.62%, respectively. The kappa agreement of WHO clinical stages was also insignificant when compared with the disaggregated CD4 counts in different categories. The correlation of WHO clinical staging was inversely associated with the CD4 count, and the magnitude of the correlation was 5.22%.

**Conclusions:**

The WHO clinical staging had high sensitivity but low specificity in predicting patients with CD4 count <200 cells/*μ*l, <350 cells/*μ*l, and <500 cells/*μ*l. There was poor correlation and agreement between CD4 T-lymphocyte count and WHO clinical staging. Therefore, WHO clinical staging alone may not provide accurate information on the immunological status of patients, and hence, it is better to use the CDC definition rather than the WHO clinical definition.

## 1. Introduction

The human immunodeficiency virus (HIV) infects and destroys CD4+ T-lymphocyte cells, which leads to persistent decline in the number of CD4+ T-lymphocyte cells and immune function as the disease progresses [1], making it difficult to fight infections. After a progressive depletion of CD4+ T-cells, the risk of opportunistic infections increases [2]. The World Health Organization (WHO) adopted a clinical staging system for HIV/AIDS in 1990, emphasizing the use of clinical parameters to guide clinical decision-making for the management of HIV-infected individuals. This system was designed mainly for use in resource-limited settings, especially Africa with limited access to laboratory services, and can be used to guide HIV care even in the absence of CD4 T-cell count [3]. The WHO system for adults sorts patients into one of four hierarchical clinical stages ranging from stage 1 (asymptomatic) to stage 4 (AIDS). Patients are assigned to a particular stage when they demonstrate at least one clinical condition in the respective stage's criteria [3].

Other clinical classifications from the Centers for Disease Control and Prevention (CDC) are based on virological, immunological, and clinical parameters which require laboratory confirmation [4]. However, WHO clinical staging is symptom based and may not correctly identify opportunistic infections [5]. On the other hand, patients may have no clinical symptoms despite low CD4 T-cell counts. Thus, if used alone, the WHO clinical stage may disqualify patients for future ART prognosis evaluations [6].

Like CD4 counts and viral load determinations, recognition of these clinical findings included in the WHO system is an important method for identifying HIV-infected individuals at high risk for morbidity and mortality. Although there are no cutoff points for CD4 T-cell count or WHO clinical stage to commence ART at this time, assessing prediction of CD4 T-lymphocyte count using WHO clinical staging among ART-naïve HIV-infected adolescents and adults could attract scientists at improving the positive predictive value of WHO clinical staging in identifying patients with immunosuppression in resource-limited settings.

There were no previous reported studies in Ethiopia which show the relationships between the revised WHO clinical staging and CD4 T-cell count. Thus, the main aim of this study was to assess predictability of CD4 T-lymphocyte count using WHO clinical staging among ART-naïve HIV-infected adolescents and adults in northern Ethiopia.

## 2. Methods and Materials

### 2.1. Study Setting and Period

The study was conducted in the Tigray Region, north Ethiopia. The Tigray Region is the 6th largest by area and the 4th most populous of the 9 Regional States of Ethiopia [7]. The Region had an estimated population of 5,055,999 in 2016. There are both public and private health facilities in the region. The public health facilities were 2 specialized hospitals, 15 general hospitals, 22 primary hospitals, 202 health centers, and 712 health posts. There are more than 500 private health care facilities including two general hospitals [8].

### 2.2. Study Design and Data Sources

A retrospective cross-sectional study was conducted from April 2015 to January 2019. The data were extracted from a database at Tigray Health Research Institute on HIV-infected patients on antiretroviral treatment for monitoring their viral load. All the people living with HIV and enrolled in ART care in Tigray health care facilities for at least 6 months whose blood sample was sent for VL determination through standard sample transportation technique to the regional laboratory/THRI from April 2015 to January 2019 were included. This study was done among 19525 patients who had complete data on WHO stage, immunological, and viral load in the THRI database.

### 2.3. Eligibility Criteria

  (i) Patients enrolled to a naïve ART care less than 15 years were excluded from the study
  (ii) Patients whose samples were determined for viral load were included in the study
  (ii) Patients who were seen and assessed their clinical and immunological status at the first diagnosis center were included in the study

### 2.4. Sampling Procedure

**Figure 1:**
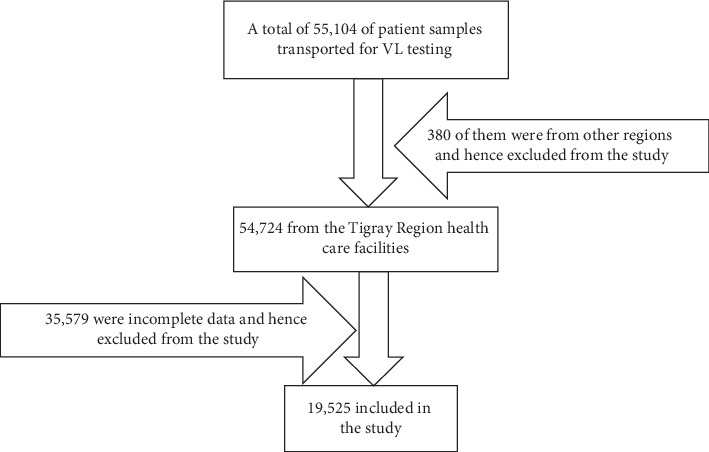
Diagrammatic presentation of the sampling procedures.

This was part of the previously published studies [9, 10]. To come up with the final sample, all necessary records of patients from the database of THRI were reviewed and then all the study participants which fulfilled the eligibility criteria were included in the study (Figure 1).

### 2.5. Data Collection Procedure

All the data in the THRI database were exported to Microsoft Excel 2013, and then data verification and filtration were done before exporting to STATA 14.0. CD4+ T-cell count was measured at the baseline in the respective health care facilities or at the nearby referral laboratory where blood samples were transported. Baseline WHO stage, baseline CD4+ T-cell count, and other characteristics of the patients were extracted from the database.

### 2.6. Data Quality Assurance

Data completeness and consistency was checked using Microsoft Excel 2013. Data cleaning was done with box plot and running frequencies for each variable in STATA version 14.0 to check outliers and inconsistencies for accuracy purpose. The normality of the continuous variables was checked by normal probability plots. CD4+ T-cell count was checked before running patient samples. Low-, medium-, and high-quality controls were checked to evaluate run validity of the machines (FACS count/Pima/FACS presto) in each of the laboratory.

### 2.7. Data Analysis

Analysis was done using STATA-14.0 to estimate the frequencies, mean, and median of CD4 T-cell count in each WHO stage. Univariate analysis was used to determine the sociodemographic and clinical characteristics of the study population. The findings were presented in different types of graphs. Kernel density estimation was used to estimate the distribution of CD4 T-lymphocyte counts in total and gender based. The median (interquartile range (IQR)) of the CD4 T-lymphocyte count was estimated for each WHO clinical stage category in total and gender based using box-and-whisker plot. The distribution of the CD4 count was also displayed using the local polynomial smoothed line with confidence interval (CI) to show possible relationships between CD4 counts and the different WHO clinical stages.

WHO clinical stages were cross-tabulated with the different categories of CD4 counts to verify their relationships using sensitivity, specificity, positive predictive value, negative predictive value, and kappa agreement. Finally, correlation was calculated to show the relationship between WHO clinical stage and CD4 counts.

## 3. Results

### 3.1. Patient Characteristics

The study was conducted on 19525 HIV-infected ART-enrolled patients. About 95.28% of the patients were in WHO clinical stages I and II. Only 4.72% of the patients were in WHO clinical stages III and IV. However, around 50% of the patients had CD4 count of less than 200 T-cells/*μ*l. About 26.62%, 12.33%, and 11.44% of the patients had CD4 count of 200–349, 350–499, and 500 T-cells/*μ*l and above, respectively (Table 1).

### 3.2. Relationship between CD4 Count and WHO Clinical Stages

**Figure 2:**
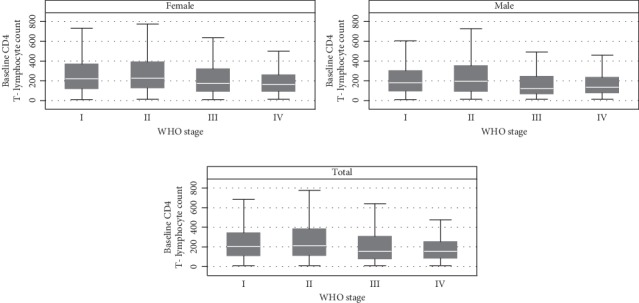
Box-and-whisker plot showing the median and range distribution of CD4+ T-lymphocyte counts at the different WHO clinical stages of HIV/AIDS among ART-naïve HIV-infected adolescents and adults in northern Ethiopia, *n* = 19525.

CD4 count was not normally distributed, and hence, the median was used to assess central tendency. The median (IQR) in overall WHO clinical stages was 201 (112–341), and the median (interquartile range (IQR)) CD4 T-cell count in the WHO clinical stages I, II, III, and IV in both males and females was 203 (114–342), 214 (115–380), 155 (81.5–306), and 156 (88–250), respectively. The median (IQR) for the WHO clinical stages I, II, III, and IV in females was 219 (123–367), 228 (130–389.5), 172 (94–318), and 164.5 (95–257), respectively, whereas the median (IQR) for the WHO stages I, II, III, and IV in males was 179 (97–300), 194 (95–349), 122 (70–242), and 135 (80–233), respectively (Figure 2).

**Figure 3:**
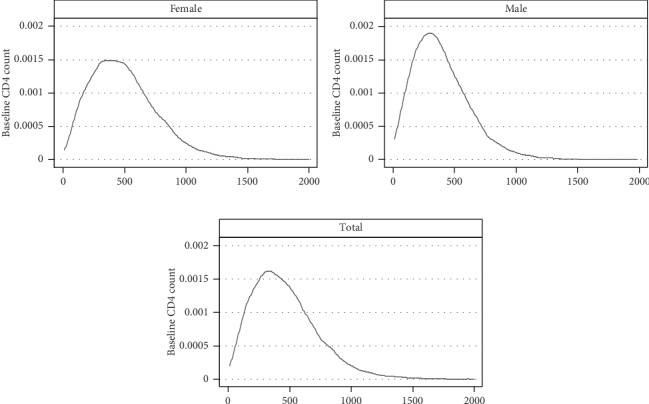
Distribution of baseline CD4 count among ART-naïve HIV-infected adolescents and adults in northern Ethiopia, *n* = 19525.

The kernel density estimation (KDE) for the CD4 T-lymphocyte count showed that most of the baseline CD-4 count observations were below 500 cells/*μ*l in total and in both genders (Figure 3). The KDE graph by gender also shows that males have lower CD4 T-lymphocyte count compared to females during the ART enrollment (Figure 3).

The cross-tabulation between WHO clinical stage and CD4 T-lymphocyte count in cells/*μ*l showed that among the patients with <200 cells/*μ*l, 90.33%, 3.83%, 2.56%, and 3.27% were in WHO clinical stages I, II, III, and IV, respectively. About 92.23%, 3.60%, 1.63%, and 2.54% of the patients with 200–349 CD4 T-cells/*μ*l were in WHO stages I, II, III, and IV, respectively. Similarly, about 92.70%, 4.57%, 1.34%, and 1.39% of the patients had 500 and above CD4 T-cells/*μ*l were in WHO stages I, II, III, and IV, respectively. About 14885 (76.24%) of the patients belonged to both advanced and severe immunosuppression categories, and the majority of 14,103 (94.75%) patients with either advanced or severe immunosuppression were in WHO clinical stages I and II (Table 2).

**Figure 4:**
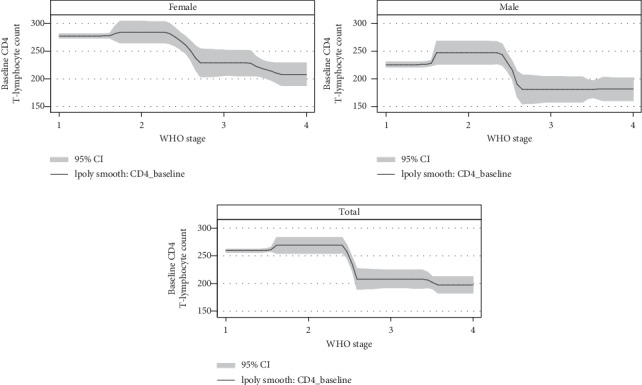
The polynomial with the 95% confidence interval of the WHO stage and baseline CD4 count among ART-naïve HIV-infected adolescents and adults in northern Ethiopia, *n* = 19525.

As shown in Figure 4, most of the observations were in WHO clinical stage I, and hence, the 95% confidence interval at the beginning of the line graph was narrow. However, it starts to widen at WHO stage II and tries to narrow in WHO stage III in total and both females and males. Compared to the total and females, males have a wider interval in all the WHO stages. The graph also shows that the CD4 T-cell count begins to decline from WHO stage II to WHO stage III rapidly. However, the decline of CD4 T-cell count tends to be a stable declination from WHO III to WHO IV (Figure 4).

WHO clinical stages were compared using the CD4 T-lymphocyte to check sensitivity, specificity, positive predictive value (PPV), negative predictive value (NPV), and agreement (the kappa test). About 95% of the patients were in WHO clinical stages I and II. Among the patients with less than 200 CD4 T-cells/*μ*l, about 94.17% were in WHO stages I and II. Only around 4% of the patients with ≥200 cells/mm^3^ CD4 count were in WHO stages III and IV (Table 3).

The sensitivity of the WHO clinical stages to predict CD4 T-cell counts of <200 cells/*μ*l was 94.17% with a specificity of 3.62%. The PPV was 49.03%, and the NPV was 3.62%, whereas the sensitivity of the WHO clinical stages to predict CD4 T-cell counts of <350 cells/*μ*l was 94.75% with a specificity of 3.00%. The PPV was 75.81%, and the NPV was 15.09%. Similarly, the sensitivity of the WHO clinical stages to predict CD4 T-cell counts of <500 cells/*μ*l was 95.03% with a specificity of 2.73%; the PPV was 88.32%, and the NPV was 6.62% (Table 3).

The kappa agreement test showed that the agreement between CD4 count and WHO clinical stages was insignificant. The agreement of WHO stages was also in significant when compared with the disaggregated CD4 counts in different categories (Table 4). Correlation testing revealed a Spearman coefficient of *r* = −0.0522 (Table 4).

The correlation of the WHO clinical stages was inversely associated with the CD4 count, and the magnitude of the correlation was 5.22% (Table 5).

## 4. Discussion

The main aim of this study was to assess the relationship between WHO clinical stages and baseline CD4 count among 19525 ART-naïve HIV-infected adolescents and adults in northern Ethiopia, using a retrospective study. Above 91% of the patients in this study were in WHO clinical stage I which is high compared to a study conducted in Kenya where 29.65 of the patients were in WHO clinical stages I and II at the time of HIV diagnosis [6]. Similarly, 76.23% of the study participants had CD4 count of less than 350 CD4 T-cells/*μ*l. A study from Kenya reported that 75% of the patients had <350 CD4 count.

Patients who were in WHO clinical stages I and II with CD4 counts below 200 cells/*μ*l accounted for 94.17% of all the patients with severe immunosuppression. Only 356 (3.62%) of the patients with CD4 counts above 200 cell counts/*μ*l were in WHO clinical stages III and IV. This study finding was lower compared to a study from Uganda which reported that more than half of the study subjects with ≥200 CD4 cells/*μ*l were in WHO stage III or IV [11]. The differences might be due to the use of different WHO staging definitions or differences in normal range CD4 counts in the general population.

In this study, an overall median (IQR) CD4 T-cell count was 201 (112–341). The median (IQR) CD4 T-cell counts were 203 (114–342), 214 (115–380), 155 (81.5–306), 156 (88–250), and 201 (112–341) in WHO clinical stages I, II, III, and IV, respectively. This was lower except in WHO clinical stage IV compared to the studies reported from Saudi Arabia [12] and Kenya [6]. Potential explanations for these differences could be that Ethiopians might have a relatively lower normal CD4 count compared to other populations [13] though it requires further validation study. As per the WHO classification [5], about 14885 (76.24%) of the patients belong to severe or advanced immunosuppression and the majority (95.28%) of the study subjects belongs to WHO clinical stages I and II. This study finding of the CD4 count category is comparable with the finding from India which reported that about 77.32% of patients had advanced or severe immunosuppression (CD4 counts < 350 cells/mm^3^). However, the findings of this study on the WHO staging are higher as compared to the Indian study which showed 73.07% of them belonged to stage I or II [14]. These differences might be attributed due to different definitions to commence ART in the countries or change of standards over time to enroll patients into ART.

The sensitivity and specificity of WHO clinical stages to predict CD4 T-cell counts of <200 cells/*μ*l were 94.17% and 3.62%, respectively. The sensitivity was higher, but the specificity was lower compared to studies from Kenya (sensitivity 53% and specificity 89%), Uganda (sensitivity 52% and specificity 68%), and Tanzania (sensitivity 75% and specificity 36%) [6, 11, 15].

The sensitivity and specificity of the WHO clinical stages to predict CD4 T-cell counts of <350 cells/*μ*l were 94.75% and 3.00%, respectively. A similar study from Uganda found that WHO clinical stages I and II have a sensitivity of 49.1% in predicting CD4 T-cell counts of 350 cells/*μ*l [16]. Another study from Kenya showed that WHO clinical stage had a sensitivity of 63% in correctly predicting CD4 counts greater than 350 cells/*μ*l and specificity of 82% in identifying patients with CD4 counts less than 350 cells/*μ*l [6]. The low specificity in this study demonstrates that most patients presenting with severe immunosuppression have been classified in WHO clinical stages I and II. This variation may be due to the clinicians' knowledge and practice variations or patient characteristic differences.

The sensitivity and specificity of the WHO clinical stages to predict CD4 T-cell counts of <500 cells/*μ*l were 95.03% and 2.73%, respectively. The kappa agreement test as well showed that CD4 count and WHO clinical stages were not statistically significantly associated. This study demonstrated that WHO clinical stages had a low specificity and was able to correctly identify only 3% of patients with CD4 counts of less than 350 cells/*μ*l. However, the sensitivity was high and missed 94.75% of patients with CD4 T-cell count of less than 350 cells/*μ*l. The sensitivity was higher but the specificity was lower compared to the studies conducted in Uganda [16] and Tanzania [15]. The low specificity could have resulted from inadequate documentation of the history, all the clinical symptoms, and examination findings.

The correlation of WHO clinical stages with CD4 T-cells count was revealed, *r* = −0.0522. This is much lower compared to a study conducted in Saudi Arabia, which reported a correlation of *r* = –0.65 [12]. The variations might be attributed to sample size as small sample size decreases the power of the study, or it could be due to the variations on providing quality ART service among the countries.

### 4.1. Strength and Limitations of the Study

This study was not without limitation. The accuracy of the clinical staging by the health care providers is not known. This might be the reason why most of the patients were classified in WHO stage I. Despite these limitations, this study was done on a large sample size with appropriate statistical analysis techniques that provide important information regarding the relationships between WHO stages and CD4 T-lymphocyte count.

## 5. Conclusions

The WHO clinical stages had high sensitivity but low specificity in predicting patients with CD4 count <200 cells/*μ*l, <350 cells/*μ*l, and <500 cells/*μ*l. There was poor correlation and agreement between CD4 T-lymphocyte count and WHO clinical staging. Therefore, WHO clinical staging alone may not provide accurate information on the immunological status of patients, and hence, it is better to use the CDC definition rather than the WHO clinical definition.

## Figures and Tables

**Table 1:** Characteristics of patients in terms of WHO clinical stage and CD4 count among ART-naïve HIV-infected adolescents and adults in northern Ethiopia.

Variable	Category	Frequency	Percentage
WHO stage	Stage I	17,829	91.31
	Stage II	775	3.97
	Stage III	408	2.09
	Stage IV	513	2.63

CD4+ T-lymphocyte cell counts/*μ*l	<200	9,687	49.61
	200–349	5,198	26.62
	350–499	2,407	12.33
	500 and above	2,233	11.44

**Table 2:** Cross-tabulation of the WHO stages with CD4 T-lymphocyte cell count among ART-naïve HIV-infected adolescents and adults in northern Ethiopia, *n* = 19525.

WHO stage	CD4 T-lymphocyte count/μl				Total *n* (%)
	Severe immunosuppression *n* (%)	Advanced immunosuppression *n* (%)	Mild immunosuppression *n* (%)	Not significant immunosuppression *n* (%)	
I	8,750 (90.33)	4,794 (92.23)	2,215 (92.02)	2,070 (92.70)	17,829 (91.31)
II	372 (3.84)	187 (3.60)	114 (4.74)	102 (4.57)	775 (3.97)
III	248 (2.56)	85 (1.63)	45 (1.87)	30 (1.34)	408 (2.09)
IV	317 (3.27)	132 (2.54)	33 (1.37)	31 (1.39)	513 (2.63)
Total	9,687 (100)	5,198 (100)	2,407 (100)	2,233 (100)	19,525 (100)

Severe immunosuppression: <200 CD4 T-lymphocyte cell counts/*μ*l. Advanced immunosuppression: 200–349 CD4 T-lymphocyte cells/*μ*l. Mild immunosuppression: 350–499 CD4 T-lymphocyte cells/*μ*l. Not significant immunosuppression: ≥500 T-lymphocyte cells/*μ*l.

**Table 3:** The relationship between CD4 T-lymphocyte count and WHO stages among ART-naïve HIV-infected adolescents and adults in northern Ethiopia, *n* = 19525.

WHO stage	CD4 T-lymphocyte count		Total
	<200 cell/*μ*l	≥200 cell/*μ*l	
I and II	9,122 (94.17)	9482 (96.38)	18,604 (95.28)
III and IV	565 (5.83)	356 (3.62)	921 (4.72)
Total	9,687 (100.00)	9,838 (100.00)	19,525 (100.00)
Sensitivity	94.17%		
Specificity	3.62%		
Positive predictive value (PPV)	49.03%		
Negative predictive value (NPV)	3.62%		

	<350 cell/mm^3^	≥350 cell/mm^3^	
I and II	14,103 (94.75)	4501 (97.00)	18604 (95.28)
III and IV	782 (5.25)	139 (3.00)	921 (4.72)
Total	14,885 (100.00)	4,640 (100.00)	19,525 (100.00)
Sensitivity	94.75%		
Specificity	3.00%		
Positive predictive value (PPV)	75.81%		
Negative predictive value (NPV)	15.09		

	<500 cell/mm^3^	≥500 cell/mm^3^	
I and II	16432 (95.03)	2172 (97.27)	18604 (95.28)
III and IV	860 (4.97)	61 (2.73)	921 (4.72)
Total	17,292 (100.00)	2,233 (100.00)	19,525 (100.000)
Sensitivity	95.03%		
Specificity	2.73%		
Positive predictive value (PPV)	88.32%		
Negative predictive value (NPV)	6.62%		

**Table 4:** Kappa test of the WHO clinical stage with different CD4 count categories among ART-naïve HIV-infected adolescents and adults in northern Ethiopia, *n* = 19525.

Kappa test	Agreement (%)	Expected agreement (%)	Kappa	Std. Err.	Z	Prob > Z
CD4 count with WHO stages	46.16	46.92	−0.0143	0.0029	−4.89	1.0000
CD4 count <200 and ≥200 cells/mm^3^ with WHO stages	46.88	47.30	−0.0081	0.0030	−2.73	0.9968
CD4 count <350 and ≥350 cells/mm^3^ with WHO stage	70.47	70.56	−0.0028	0.0045	−0.63	0.7343
CD4 count <500 and ≥500 cells/mm^3^ with WHO stage	81.23	81.32	−0.0048	0.0053	−0.91	0.8181

**Table 5:** The correlation between the various WHO clinical stages of HIV/AIDS and CD4+ T-lymphocyte counts among ART-naïve HIV-infected adolescents and adults in northern Ethiopia, *n* = 19525.

Correlation	CD4 T-lymphocyte count	WHO stage
CD4 T-lymphocyte count	1.0000	
WHO stage	−0.0522	1.0000

## Data Availability

The data that support the findings of this study are available from Tigray Health Research Institute, but restrictions apply to the availability of these data, which were used under license for the current study, and so they are not publicly available. Data are, however, available from the authors upon reasonable request and with permission of Tigray Health Research Institute Institution Review Board. Please direct data access requests to the Tigray Health Research Institute (Tel: +251342413648, Fax: +2551342414918, e-mail: healthresearchinstitutetigray@gmail.com/ institutional.rereview.board.thri@gmail.com, P.o.box: 1547, Mekelle, Tigray, Ethiopia).
